# Pharmacokinetics and nephrotoxicity of cisplatin modulated by combination therapy with brusatol

**DOI:** 10.3389/fphar.2026.1708101

**Published:** 2026-02-09

**Authors:** Nan Guo, Yahui Zhang, Guiyan Yuan, Xiaoran Zhang, Wen Zhang, Qing Wen

**Affiliations:** 1 Department of Pharmacy, Shandong Provincial Hospital Affiliated to Shandong First Medical University, Jinan, China; 2 Phase I Drug Clinical Trial Center, Qilu Hospital of Shandong University, Jinan, China; 3 Clinical Research Center, Central Hospital Affiliated to Shandong First Medical University, Jinan, China

**Keywords:** brusatol, cisplatin, drug interaction, HPLC-MS, nephrotoxicity

## Abstract

**Ethnopharmacological Relevance:**

Quassinoid brusatol, isolated from the traditional Chinese medicine *Brucea javanica (L.) Merr.*, significantly increases the intracellular concentration of cisplatin and reduces tumor size by inhibiting the Nrf2 pathway. However, the underlying mechanism remains unclear.

**Objective:**

This study focuses on the effects of brusatol on plasma pharmacokinetics, tissue distribution, and nephrotoxicity of cisplatin.

**Materials and Methods:**

For the pharmacokinetic study, 120 Kunming mice were randomly assigned to receive cisplatin (10 mg/kg) alone or in combination with brusatol (2 mg/kg). Blood and tissue samples were collected to evaluate the *in vivo* pharmacokinetic interaction. In the nephrotoxicity study, 30 Kunming mice were randomly divided into groups to receive cisplatin (10 mg/kg) alone or brusatol pretreatment (1 mg/kg or 2 mg/kg). Two control groups received brusatol (1 mg/kg) or solvent. Blood samples and kidney tissues were collected to investigate the underlying mechanisms of toxicity.

**Results:**

The developed HPLC-MS method demonstrated high precision and accuracy, meeting the requirements for biological sample analysis. Brusatol reduced the plasma concentration of cisplatin, increased the apparent volume of distribution (Vd), and significantly increased cisplatin concentrations in the kidney and lung, particularly in the kidney. Combined with brusatol, serum levels of blood urea nitrogen and creatinine increased, antioxidant indices in kidney tissue decreased, and inflammatory factors increased. Pathological findings revealed increased tubular congestion.

**Discussion and Conclusion:**

Brusatol increases renal cisplatin concentrations by altering its pharmacokinetics, intensifying dose-dependent nephrotoxicity. Further development as a chemotherapy sensitizer requires understanding the mechanisms of this effect and optimizing the combined dose.

## 1 Introduction

Molecular targeted therapy and immunotherapy have significantly prolonged the overall survival of patients with cancer but are often associated with an increased risk of adverse reactions. Many patients use Traditional Chinese Medicine (TCM) as a complementary treatment to alleviate these adverse effects and improve therapeutic efficacy. However, predicting herb-drug interactions remains challenging, and ensuring the safety of such combinations remains challenging.

The quassinoid brusatol, isolated from the traditional Chinese medicine *Brucea javanica (L.) Merr.* (Hall et al., 1979), possesses diverse pharmacological effects, including anticancer, antimalarial, and antiviral properties (Wright et al., 1993; Yan et al., 2010). Many studies have explored the antitumor mechanisms of quassinoid brusatol, revealing that brusatol inhibits protein synthesis, activates the nuclear translocation of NF-kB, and downregulates the protein levels of the proto-oncogene C-MYC (Cuendet et al., 2004). However, in 2011, Ren et al. reported contrasting findings, indicating that brusatol alone does not demonstrate anticancer activity but effectively inhibits the Nrf2 pathway and improves the efficacy of cisplatin(CDDP). Brusatol inhibits the Nrf2 pathway by enhancing the ubiquitination and degradation of the Nrf2 protein, thereby downregulating the protein levels of Nrf2. This inhibitory effect operates independently of Keap1 and proteasome activity and does not affect the mRNA levels of Nrf2. The combination of CDDP and brusatol was shown to significantly downregulate the proliferation of A549 cancer cells and tumor volume in mouse models with tumors (Ren et al., 2011). These findings redirected the research focus to brusatol, highlighting its potential as an adjunctive agent for various cancers, including nonsmall cell lung cancer (NSCLC) and kidney cancer (Yang et al., 2020; Wang et al., 2022; Xing et al., 2022). Therefore, brusatol has emerged as a promising candidate for future therapeutic drug development.

Despite these advances, the mechanisms underlying the therapeutic effects of brusatol remain unclear. Brusatol inhibits the Nrf2 pathway in tumor cells, increasing tumor tissue sensitivity to chemotherapeutic agents (Wang et al., 2022). However, its non-specific inhibitory effects can also sensitize non-target tissues to chemotherapy, potentially increasing the risk of toxicity alongside efficacy. Some studies have reported significant weight loss in mice treated with the combination of brusatol and chemotherapy, suggesting increased toxicity (Tao et al., 2014). Adverse reactions to chemotherapeutic drugs, such as CDDP, are often dose-dependent. Therefore, exploring the “concentration-effect-toxicity” relationship of brusatol combined with chemotherapeutic agents is critical for drug development. This study investigates the effects of brusatol on the pharmacokinetics and nephrotoxicity of CDDP using HPLC-MS/MS to promote its development as an adjuvant chemotherapeutic drug.

## 2 Materials and methods

### 2.1 Drugs

The Cisplatin (CDDP) standard (purity 98.00%, catalog number: 15,663–27–1) was purchased from TargetMol, while the injection-grade CDDP powder was obtained from QiLu Company (Shandong, China, catalog number: FA4A8046A). The reference standard for brusatol (purity >97%, catalog number: 180,306) was procured from Shanghai Tauto Biotech Co., Ltd. Sodium diethyldithiocarbamate trihydrate (DDTC) was obtained from Aladdin Industrial Corporation (catalog number: E1823148), and carbamazepine was provided by the National Institutes for Food and Drug Control (catalog number: 100,142–201105). The detection kits for glutathione (GSH), superoxide dismutase (SOD), catalase (CAT), malondialdehyde (MDA), nitric oxide (NO), and nitric oxide synthase (NOS) were all purchased from the Nanjing Jiancheng Bioengineering Institute (Nanjing, China, catalog numbers: MPC 240986, MPC 20241011).

### 2.2 Animals

The Kunming mice provided by SPF (Beijing) Biotechnology Co., Ltd. were selected for the pharmacokinetic (PK) and nephrotoxicity studies. All animal experiments were conducted strictly compliance with relevant national regulations, local guidelines, and the ARRIVE 2.0 guidelines. The study was approved by the Animal Ethics Committee of Shandong Provincial Hospital Affiliated with Shandong First Medical University (ethics approval number: 2023–656). Before the experiments, all animals were acclimatized for 1 week under standard environmental conditions with free access to food and water. Blood samples were collected from the posterior orbital venous plexus under 2% isoflurane inhalation anesthesia. Thereafter, cervical dislocation was conducted to collect tissue samples.

For the PK study, 90 male mice (20–25 g) were randomly divided into two groups: the CDDP group and the combination group. CDDP (10 mg/kg) was intraperitoneally injected into mice in the CDDP group. In comparison, those in the combination group received brusatol (2 mg/kg) via tail vein injection 30 min before administering CDDP. Blood and tissue samples were collected at 5 min, 15 min, 30 min, 1 h, 2 h, 4 h, 8 h, 12 h, and 24 h after injecting CDDP.

For the nephrotoxicity studies, 30 male mice were randomly divided into five groups. From day 1 to day 3, mice in the BRU group and the combination treatment group received BRU via their tail veins (1 mg/kg for both the BRU and low-dose combination groups and 2 mg/kg for the high-dose combination group). In contrast, mice in the blank control and CDDP groups received an equivalent volume of BRU blank solvent (0.9% saline containing 1% DMSO). From day 4 to day 6, mice in the CDDP group and the combination treatment group received CDDP at a dose of 10 mg/kg. Meanwhile, mice in the blank control and BRU groups received an equal volume of saline.

Blood samples were collected 2 days after the final dose to assess blood urea nitrogen (BUN) and creatinine (CR) levels. Kidney tissues were harvested to measure the activities of glutathione (GSH), superoxide dismutase (SOD), catalase (CAT), malondialdehyde (MDA), nitric oxide (NO), and nitric oxide synthase (NOS). Histopathological changes were evaluated using hematoxylin-eosin (HE) staining.

### 2.3 PK and tissue distribution studies of CDDP in mice

First, 100 μL of plasma sample was spiked with 10 μL of the internal standard (IS) solution and 20 μL of 0.1 M NaOH (1% DDTC). After vortexing for 2 min, the mixture was incubated in a 40 °C water bath for 30 min. Next, 500 µL of a mixture of ethyl acetate and n-hexane (1:1) was added. Following an additional vortexing for 2 min, the sample was centrifuged at 10,800 rpm for 5 min. Then, 400 µL of the supernatant was transferred to a clean EP tube. After nitrogen evaporation, the residue was dissolved in 100 µL of acetonitrile-water (75:25). The tissue samples were weighed and homogenized in nine volumes of 0.9% saline. The resulting supernatant was collected and processed using the same preparation method as for the plasma samples.

CDDP detection was conducted using an Agilent 1,100 series HPLC system coupled with an Agilent 1,100 series G1946D mass spectrometer (Agilent Technologies, United States) equipped with an electrospray ionization (ESI) source operating in positive ion mode. Chromatographic separation was achieved on an Intersil ODS-3 column (5 μm, 4.6 × 150 mm, GL Sciences Inc., Japan) maintained at 25 °C. The mobile phase consisted of acetonitrile and 0.1% formic acid (70:30, v/v) at a flow rate of 0.5 mL/min, with an injection volume of 5 μL.

Mass spectrometry was conducted in an ESI-positive ion mode. The spray gas pressure was set at 50 psig, the nitrogen gas flow rate was 11.0 L/min, and the capillary voltage was 4000 V. The fragmentation voltages was 150 V for CDDP and 110 V for IS. Quantification was conducted in the selected ion monitoring (SIM) mode, with target ionsidentified at m/z 640.2 for CDDP and m/z 237.0 for IS.

The method was validated following the guidelines of bioanalytical method validation provided by the US Food and Drug Administration (US Department of Health and Human Services 2022) and the China Food and Drug Administration (China Food and Drug Administration [CFDA] 2015). Specifically, we considered selectivity, matrix effect, linearity, recovery, accuracy and precision, dilution integrity, and stability evaluations.

### 2.4 Effect of brusatol on CDDP-induced acute nephrotoxicity in mice

Biochemical assays were conducted using an automatic chemistry analyzer (Shenzhen Leidu Life Technology, Chemray240). The plasma levels of BUN and CR were measured to determine kidney function. Kidney samples were homogenized at 4 °C and centrifuged at 3,000 rpm for 20 min. The resulting supernatants were collected to measure the levels of MDA and GSH and the activities of SOD, CAT, NO, and NOS in kidney homogenates. Following the manufacturer’s protocols for each kit, we sequentially added the reagents included in the respective kits, measured the absorbance at the specified wavelength, and subsequently calculated their concentration or activity.

Kidney tissues were fixed in 4% paraformaldehyde, dehydrated through an ascending series of alcohols, cleared in xylene, and embedded in paraffin using standard laboratory procedures. Paraffin-embedded specimens were sectioned and stained with hematoxylin and eosin (H&E). Histological features were observed using a light microscope (Servicebio LG-FS80), and images were captured using a panoramic scanner (3D HISTECH Panoramic MIDI, Hungary).

### 2.5 Statistical analysis

HPLC-MS data were analyzed using Agilent ChemStation software (Version B 04.03). The PK parameters of CDDP for each group were calculated using DAS 2.0. Statistical analyses were conducted with SPSS 25.0 (IBM Corporation, Chicago, IL) and GraphPad Prism 9.0. Statistical significance was assessed using ANOVA and Student’s t-test, with a P value <0.05 deemed statistically significant.

## 3 Results

### 3.1 Method validation

The calibration curve for CDDP was linear within the range of 5–3,000 ng/mL in plasma and 5–1,200 ng/mL in tissue (*R* ^2^ > 0.99). CDDP extraction recoveries were evaluated at two quality control (QC) levels (QC-L and QC-H) in plasma (53.64% ± 4.33% and 54.02% ± 1.18%), kidneys (63.01% ± 6.21% and 62.67% ± 3.63%), and lung (58.56% ± 6.86% and 62.77% ± 1.80%), respectively. For all three matrices, the relative standard deviation (RSD) of the matrix effect normalization factor for CDDP was <15%, suggesting that the matrix effect of endogenous substances was negligible in this analytical method.

Precision and accuracy were assessed by analyzing QC samples and low quantification (LLOQ) samples with five replicates on the same day (intraday) and across three consecutive days (interday). The precision and accuracy in plasma, kidney, and lung sampleswere within the acceptable ranges (Table 1).

**TABLE 1:** The precision and accuracy of CDDP in plasma, kidneys, and lungs (n = 5).

Sample	Concentration	Intra-day		Inter-day	
	(ng/mL)	Mean ± SD	RSD (%)	Mean ± SD	RSD (%)
Plasma	5	5.018 ± 0.414	8.151	5.086 ± 0.489	9.6
	10	9.823 ± 0.977	9.946	9.949 ± 8.2	8.2
	800	794.608 ± 69.967	8.805	787.965 ± 55.777	7.1
	2,500	2412.368 ± 216.909	8.992	2469.980 ± 206.101	8.3
Kidneys	5	5.032 ± 0.58	11.47	5.069 ± 0.49	9.60
	8	8.005 ± 0.75	9.42	8.095 ± 0.67	8.29
	500	496.327 ± 51.16	10.31	495.506 ± 46.06	9.30
	1,000	950.612 ± 64.18	6.75	961.210 ± 48.94	5.09
Lungs	5	5.057 ± 0.52	10.21	4.915 ± 0.53	10.74
	8	8.264 ± 0.77	9.27	8.164 ± 0.54	6.61
	500	519.717 ± 32.25	6.20	506.278 ± 42.71	8.44
	1,000	978.204 ± 57.96	5.93	949.635 ± 56.44	5.94

**FIGURE 1:**
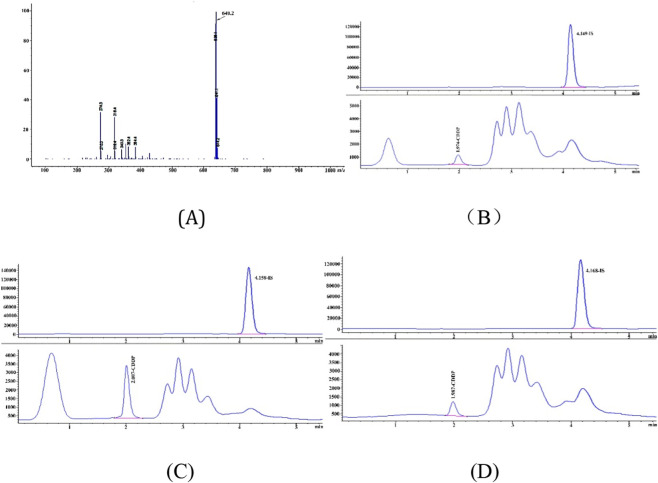
The mass spectrum **(A)** of CDDP and DDTC complex. The derivatization of CDDP with DDTC led to the formation of a Pt-DDTC derivative with a mass-to-charge ratio (m/z) of 640.0. In positive ion mode scanning, the response for [Pt-DDTC3 + H]+ was stable, and the peak shape was well-defined. Thus, m/z 640.0 was selected as the detection ion for CDDP. The typical chromatographs of CDDP (LLOQ) and the internal standard (IS) in plasma **(B)**, kidneys **(C)**, and lungs **(D)** were determined.

The stability of CDDP in plasma, kidney, and lung samples was evaluated at low and high QC levels under various conditions: storage at room temperature for 6 h, storage at −20 °C for 1, 3, and 21 days, two freeze-thaw cycles, and post-extraction storage in the autosampler at 24 °C for 12 h. The results showed that the detected concentrations of CDDP ranged between 85% and 115% of the nominal concentrations, demonstrating acceptable stability under these conditions (Figure 1).

### 3.2 Effect of pretreatment with brusatol on the pharmacokinetics of CDDP

**FIGURE 2:**
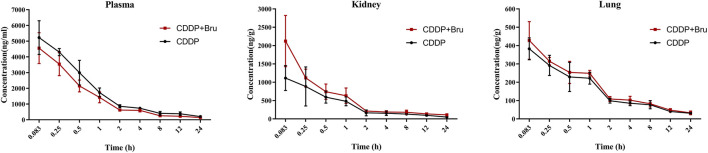
Mean concentration-time curves for CDDP (10 mg/kg) in plasma, kidneys, and lungs after intraperitoneal injection in mice in the presence or absence of treatment with brusatol (2 mg/kg) (mean ± SD, n = 5).

After a single dose of 10 mg/kg CDDP or its combination with 2 mg/kg brusatol, plasma samples from mice were analyzed using the validated method. The AUC_0-t_ of CDDP in the plasma samples of the combined group (4784.05 ng·h/mL) was significantly lower than that in the CDDP group (5329.68 ng·h/mL) (Figure 2). However, no significant alteration was observed in the elimination half-life (t_1_/_2_). The CDDP clearance rate in plasma and the apparent volume of distribution increasedin the combined group, suggesting that a greater proportion of the drug was distributed in tissues. This redistribution may reduce the hematologic toxicities of CDDP.

The tissue concentration of CDDP in tissues was calculated using the following formula: C_f_ = C_0_×V/W, where C_f_ (ng/g) is the tissue concentration, C_0_ (ng/mL) is the concentration measured by LC-MS, V (mL) is the homogenate volume and W (g) is the tissue weight.

The AUC_0-t_ of CDDP in the kidney and lung was higher after the combined use of brusatol compared to CDDP alone. The C_max_ of CDDP increased from 1,132.45 ng/g to 2122.68 ng/g in the kidney (p < 0.05) and from 390.85 ng/g to 428.06 ng/g in the lung(Table 2).

**TABLE 2:** CDDP (10 mg/kg) after intraperitoneal injection in mice in the absence or presence of treatment with brusatol (2 mg/kg) (mean ± SD, n = 5).

Group		AUC(0-t) (mg/L*h)	C_max_ (mg/L)	T_1/2z_ (h)	Cl_z_ (mL/h/kg)	V_z_ (L/kg)
Plasma	CDDP	13,538.37 ± 919.94*	5329.68 ± 1021.07	9.39 ± 2.51	0.62 ± 0.04*	0.0084 ± 0.002*
	CDDP + BRU	9113.58 ± 1462.47	4784.05 ± 807.54	9.94 ± 1.70	0.92 ± 0.14	0.0130 ± 0.0012
Kidney	CDDP	2717.86 ± 520.61*	1132 ± 361.82*	5.35 ± 1.73	3.16 ± 0.38*	0.024 ± 0.005
	CDDP + BRU	4350.55 ± 1395.91	2122.68 ± 780.39	8.66 ± 3.89	1.97 ± 0.71	0.022 ± 0.0066
Lung	CDDP	1177.96 ± 160.75	390.86 ± 60.26	5.84 ± 1.34	6.66 ± 0.88	0.056 ± 0.016
	CDDP + BRU	1238.879 ± 117.56	428.06 ± 114.86	5.55 ± 1.27	5.92 ± 1.09	0.046 ± 0.0068

* P < 0.05.

### 3.3 Effect of brusatol on CDDP -induced nephrotoxicity in mice

#### 3.3.1 Body weight

**FIGURE 3:**
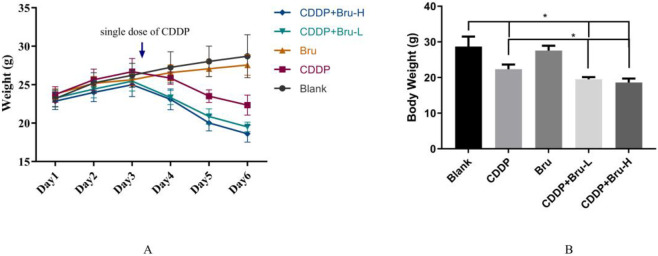
The body weight of the mice. **(A)** The Trend Chart of Body Weight Changes in Mice (day1 to day 6). **(B)** The body weight of mice before sacrifice (The body weight of mice in the cisplatin group and the combination therapy group was significantly lower than that in the blank control group; body weight in the combination therapy group was further reduced compared to the cisplatin group.) (*P < 0.05).

Body weight was recorded daily from the first day of administration. After intraperitoneal injection of CDDP, the body weight of mice significantly decreased in the CDDP and combination groups. In contrast, mice in the brusatol and blank groups showed a steady growth pattern. The body weights of the mice treated with combination therapy was even lower than the body weight of mice in the CDDP group (Figure 3). Body weights were also compared before sacrifice to further assess effect of the treatments.

#### 3.3.2 Serum BUN and CR levels

**FIGURE 4:**
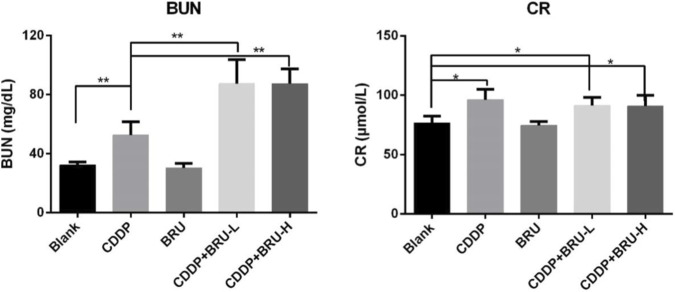
The serum levels of blood urea nitrogen (BUN) and creatinine (CR) in mice (mean ± SD) (*P < 0.05,**P < 0.01) (The levels of BUN and CR in both the cisplatin group and the combination drug group were significantly higher than those in the blank control group, with the most pronounced elevation observed in the combination drug group. The increase in BUN was more substantial compared to that in CR).

As biochemical markers of nephrotoxicity, CDDP significantly increased the serum levels of BUN (from 32.34 mg/dL to 52.79 mg/dL) and creatinine (from 76.74 μmol/L to 96.15 μmol/L) compared to the control and brusatol groups. No significant differences were observed between the brusatol and control groups (P = 0.932, P = 0.999), suggesting that brusatol does not affect kidney function. However, treatment with brusatol in combination with CDDP further increased the serum levels of BUN and creatinine compared to treatment with CDDP alone (Figure 4). These findings suggest that pretreatment with brusatol may exacerbate CDDP-induced nephrotoxicity.

#### 3.3.3 Antioxidant index

Oxidative stress is a key mechanism underlying CDDP-induced nephrotoxicity. The serum levels of GSH, SOD, CAT, MDA, NO, and NOS were measured to evaluate the effects of brusatol, CDDP, and their combination on lipid peroxidation biomarkers.

**FIGURE 5:**
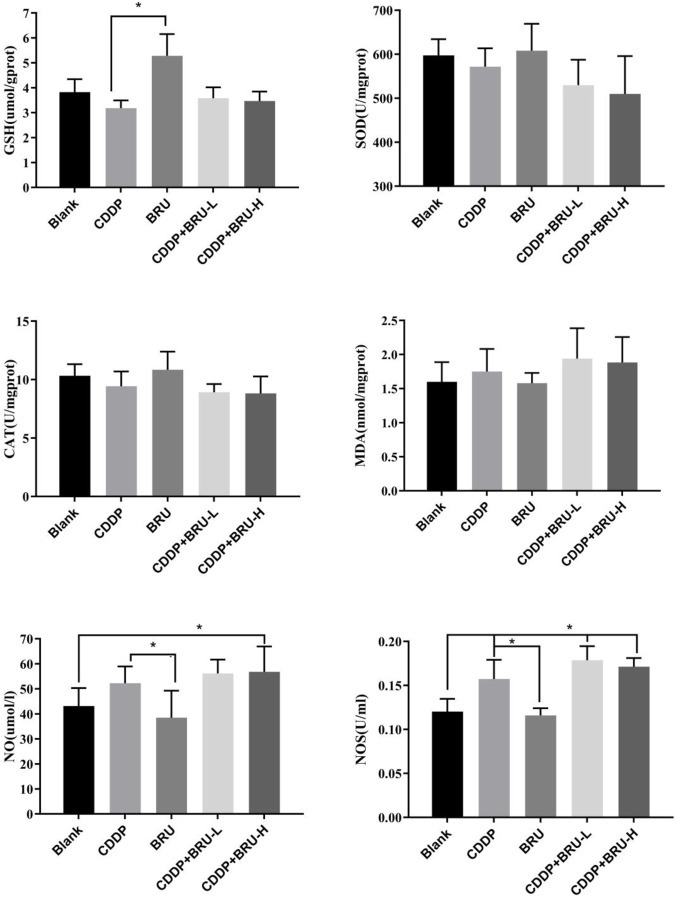
Effect of brusatol and/or CDDP on oxidative stress in the kidney (mean ± SD) (*P < 0.05,**P < 0.01) (The GSH level in the BRU group of mice was significantly elevated by 38.21% compared to that in the blank control group; the NOS activity of mice was increased by 30.87% (in the CDDP group) and 48.51% and 42.42% (in the combination group) relative to that of blank control group, compared with CDDP group, the NOS activity further increased 13.48% and 8.83% in the combination group; the NO generation in the CDDP group was increased by 21.13% compared to the blank control group, and which elevated by 30.25% and 31.72% in the combined medication group).

Compared to the control group, CDDP significantly increased MDA levels in the kidney by 9.43% while decreasing GSH, SOD, and CAT levels by 16.81%, 4.33%, and 8.74%, respectively (Figure 5). Treatment with brusatol alone significantly increased (38.21%) GSH levels compared to the control group, with no significant changes observed in SOD, CAT, or MDA levels. Brusatol alone did not induce visible apoptosis, suggesting its antioxidative properties may offer protective effects.

When brusatol was administered before CDDP, there was a slight but non-significant increase in GSH levels, restoring CDDP-induced decrease in GSH levels to normal levels seen in the control group. Additionally, compared to the CDDP group, SOD and CAT levels further decreased in the combination therapy group, while MDA levels were elevated.

After administering CDDP, NOS activity increased by 30.87% (P < 0.05), and NO generation increased by 21.13%, indicating oxidative damage caused by CDDP. In the combination group, NOS activity and NO levels further increased compared to the CDDP group, and the extent of the increase was not correlated with the brusatol dosage.

These findings suggest that while brusatol, alone, can downregulate reactive oxygen species (ROS) production in the kidney, its combination with CDDP significantly increases ROS levels,The effects of brusatol combined with CDDP exceeded those observed with CDDP alone. Increased ROS generation may be due to the increased tissue concentration of CDDP.

#### 3.3.4 Pathological changes

**FIGURE 6:**
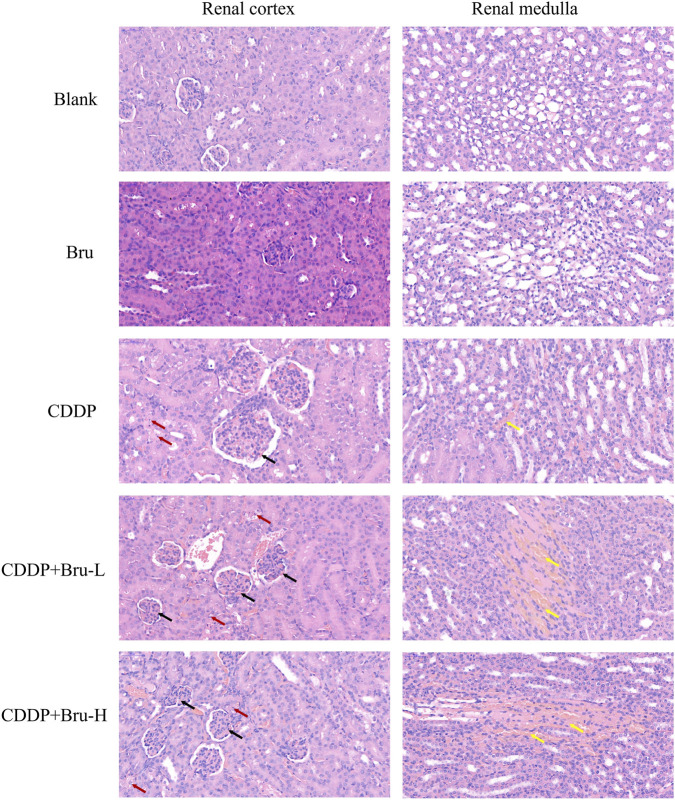
Effect of CDDP and the combination of CDDP and brusatol on histological changes in renal tissues (40×field of vision). Red arrows show karyopyknosis, hyperchromatic nuclei, and nuclear fragmentation, which suggest necrosis and apoptosis of renal tubular epithelial cells. Black arrows indicate congestion or luminal erythrocytes. Yellow arrows signify congestion in the tubulointerstitial regions. (The histological evaluation section requires blind analysis conducted by pathology experts Professor Zhang Ya.).

Light microscopic examination of renal tissue sections from the control group and brusatol group revealed normal histological structural features, while severe kidney damage was observed in the CDDP group. Histological damage was further exacerbated in thecombination therapy group (Figure 6).

In the control and brusatol groups, the renal tissue showed intact structural integrity, with normal glomerular and tubular architecture. In contrast, the CDDP group showed pronounced histological alterations, including congestion of the glomerular capillaries and erythrocytes in the lumen (indicated by black arrows). Necrosis and apoptosis of renal tubular epithelial cells were evident, characterized by karyopyknosis, hyperchromatic nuclei, or nuclear fragmentation (indicated by red arrows). Additionally, congestion was visible in the tubulointerstitial regions of the cortex and medulla (indicated by yellow arrows).

More severe capillary congestion was observed in the CDDP + BRU-L and CDDP + BRU-H groups compared to the CDDP group. However, no obviously change was observed in histological damage between the two combination groups.

## 4 Discussion

A reliable method is necessary for detecting the plasma and tissue concentrations of CDDP and investigating the PK interaction between CDDP and brusatol. Previous studies on the pharmacokinetics of CDDP in humans and animals using HPLC or HPLC-MS/MS methods mainly focused on plasma and urine samples. Specifically, few reports have adopted detection methods for assessing tissue concentrations, and there are limited studies on method validation (Guo et al., 2017; Shaik et al., 2017). Some studies have employed specialized ion exchange columns or post-column derivatization, increasing the complexity of the analytical process (El-Khateeb et al., 1999). Although ICP-MS demonstrates high sensitivity for detecting drugs containing metal elements such as CDDP, its application in clinical settings is limited by high background noise from endogenous and exogenous compounds (Zhang et al., 2015).

The HPLC-MS method developed in this study offers high sensitivity, efficiency, and ease of operation, simultaneously measuring CDDP concentrations in plasma samples and tissue homogenates. DDTC was used as a derivatizing reagent to react with CDDP before separation on an Intersil ODS-3 column. The method’s was comprehensively validated following the requirements of the National Medical Products Administration (NMPA) for biological sample analysis. All results fell within the acceptable range, supporting the reliability and robustness of the method.

Following intraperitoneal administration of CDDP (10 mg/kg), the peak plasma concentration (Cmax) reached 5,326.68 ng/mL, The concentration of CDDP rapidly decreased within 2 hours, followed by a slower elimination phase. With brusatol pretreatment, the C_max_ of CDDP decreased to 4,784.05 ng/mL in plasma. This alteration was accompanied by a significant decrease in the AUC_0-t_ in plasma and an increase in the volume of distribution, suggesting greater tissue distribution of the drug. This hypothesis was confirmed by tissue concentration analysis, showing increased levels of CDDP in the lungs and kidneys of mice in the combination treatment group. Previous studies have shown that brusatol accumulates mostly in the lungs (Guo et al., 2017); however, the increased in the concentration of CDDP in the lungs was significantly milder than the increase in the concentration of CDDPin the kidneys. These findings suggest that brusatol improves CDDP uptake in various tissues with differing sensitivities.

The ability of brusatol to specifically inhibit Nrf2 increases tumor cell sensitivity to antitumor drugs and increases drug concentrations in the intracellular space (Ren et al., 2011). Our *in vivo* studies confirmed that brusatol significantly increasesthe tissue concentrations of CDDP, particularly in the kidneys, which may intensify CDDP-induced nephrotoxicity. Cisplatin remains a cornerstone agent in the treatment of lung tumors. The observed increase in cisplatin concentration within lung tissue in the combination therapy group indicates promising therapeutic potential for Brusatol in the management of lung cancer.

Compared to treatment with CDDP alone, body weight significantly decreased in the combination treatment group. Interestingly, in the study conducted by Ren et al., tumor-bearing nude mice treated with a combination of CDDP (2 mg/kg) and brusatol (2 mg/kg) did not show significant weight loss in the initial days of treatment; however, prolonged treatment led to mild weight loss in the combination group (Ren et al., 2011). This disparity may be attributed to differences in dosage selection of CDDP. In the present study, cisplatin was administered at a dose of 10 mg/kg, which is higher than that used in the study by Ren et al. These findings further suggest that an optimal dosage regimen is critical for achieving the detoxifying and potentiating effects of brusatol.

According to the literature, CDDP induces lipid peroxidation and generates free radicals, resulting in renal tubular and glomerular injury, nephrotoxicity, and elevated levels of CR and BUN levels (Tang C. et al., 2023; Motwani et al., 2022). Our study indicated that the combination of CDDP and brusatol can increase BUN and serum creatinine levels compared to CDDP alone. Histological examination revealed more severe renal tubular epithelial cell necrosis and medullary congestion in the combined treatment group than in the CDDP group. This finding indicates that brusatol pretreatment exacerbated CDDP-induced acute nephrotoxicity. This effect was not associated with the dose of brusatol. Increased CDDP-induced acute nephrotoxicity after brusatol pretreatment is probably attributed to a significant increase in CDDP concentration in renal tissues, a critical factor that aggravates renal toxicity.

Maintaining cellular viability and function depends on a delicate balance between producing and eliminating ROS. Excess ROS generation under pathological conditions can lead to reversible or irreversible tissue damage, resulting in various diseases. GSH, a low molecular weight scavenger of free radicals, serves as the first line of defense against ROS, while SOD and CAT play critical roles in decomposing free radicals and maintaining intracellular stability (Lee et al., 2021; Tang Y. C. et al., 2023).

Compared to the CDDP treatment group, no significant changes were observed in GSH levels in the combination treatment group. At the same time, SOD and CAT activities decreased without statistical significance. MDA, a key indicator of lipid peroxidation, is normally upregulated after treatment with CDDP (Amirshahrokhi and Khalili, 2015). Consistent with previous findings, the combined treatment group exhibited elevated levels of MDA compared to the CDDP-only group (Xie et al., 2021); however, this increase did not meet the threshold of statistically significant. These results suggest that brusatol does not effectively mitigate CDDP-induced lipid peroxidation.

NO is a critical mediator of cytokine-induced injury (Yang, 2019). Biochemical analyses revealed elevated NO levels and NOS activity in the combination treatment group, suggesting more severe kidney injury. The inflammatory response plays a central role in CDDP-induced nephrotoxicity, with activation of the NF-κB pathway driving upregulating of pro-inflammatory cytokines and enzymes such as tumor necrosis factor-alpha (TNF-α), interleukins (ILs), NO, and inducible NOS (iNOS) (Turpaev and Welsh, 2015). This cascade contributes to renal damage. However, previous studies have shown that brusatol does not significantly affect the NF-κB pathway (Song et al., 2021).

Furthermore, the Keap1/Nrf2 system does not regulate NOS expression. The inhibitory effects of brusatol on NOS may be attributed to increased degradation of the NOS protein, thus exhibiting anti-inflammatory properties(Turpaev and Welsh, 2015). Although NOS expression in the combined treatment group was significantly higher than that in the CDDP group. This finding suggests that the ability of brusatol to degrade NOS protein cannot counteract the inflammatory cascade and CDDP-induced nephrotoxicity.

## 5 Conclusion

Brusatol increases the renal concentration of CDDP by modulating its pharmacokinetic, intensifying the dose-dependent nephrotoxic effects of CDDP. Although no significant differences in the extent of renal injury were observed across the various doses of Brusatol in this study, given its known anti-inflammatory and antioxidant properties, achieving a favorable balance between enhanced therapeutic efficacy and reduced toxicity remains a promising goal. Therefore, optimizing the dosing regimen between Brusatol and cisplatin will be a key focus of future research and a crucial step towards clinical translation. This study has several limitations. Given that the pharmacokinetic component involves not only blood collection from mice but also the harvesting of tissue organs, the animals cannot survive the procedure. Consequently, the pharmacokinetic and nephrotoxicity studies were conducted as separate experiments. Although the findings from these studies are mutually supportive, they are not directly linked. Based on the current experimental findings, subsequent investigations will be carried out in tumor-bearing mouse models to concurrently evaluate pharmacokinetics, therapeutic efficacy, and toxicity.

## Data Availability

The raw data supporting the conclusions of this article will be made available by the authors, without undue reservation.
