# Correction: Pharmacokinetics and nephrotoxicity of cisplatin modulated by combination therapy with brusatol

**DOI:** 10.3389/fphar.2026.1809629

**Published:** 2026-03-02

**Authors:** Nan Guo, Yahui Zhang, Guiyan Yuan, Xiaoran Zhang, Wen Zhang, Qing Wen

**Affiliations:** 1 Department of Pharmacy, Shandong Provincial Hospital Affiliated to Shandong First Medical University, Jinan, China; 2 Phase I Drug Clinical Trial Center, Qilu Hospital of Shandong University, Jinan, China; 3 Clinical Research Center, Central Hospital Affiliated to Shandong First Medical University, Jinan, China

**Keywords:** brusatol, cisplatin, drug interaction, HPLC-MS, nephrotoxicity

There was a mistake in Figure 2 as published. The middle panel of the figure was missing the caption ‘Kidney’. The corrected Figure 2 appears below.

**FIGURE 2:**
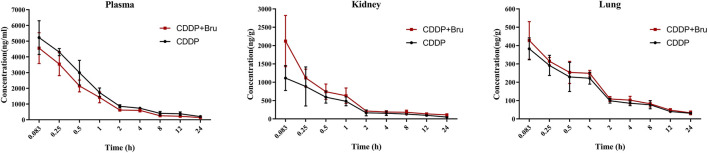
Mean concentration-time curves for CDDP (10 mg/kg) in plasma, kidneys, and lungs after intraperitoneal injection in mice in the presence or absence of treatment with brusatol (2 mg/kg) (mean ± SD, n = 5).

The original article has been updated.

